# Retraction Note: Fe_3_O_4_@SiO_2_-PMA-Cu magnetic nanoparticles as a novel catalyst for green synthesis of *β*-thiol-1,4-disubstituted-1,2,3-triazoles

**DOI:** 10.1038/s41598-025-02715-3

**Published:** 2025-05-19

**Authors:** Ronak Eisavi, Fereshteh Ahmadi

**Affiliations:** Department of Chemistry, Payame Noor Universtiy (PNU), P.O. BOX, Tehran, 19395-4697 Iran

Retraction of: *Scientific Reports* 10.1038/s41598-022-15980-3, published online 13 July 2022

The Editors have retracted this Article.

After publication of this Article, concerns were raised regarding some image irregularities and methodology discrepancies. Specifically:

• The FT-IR spectra in Figure 4 appears to originate from different sources and to have misaligned peaks relative to the X axis.

• The XRD patterns in Figure 5 appear to have highly similar noise patterns between trace a and trace b.

• Figures 7 and 13 do not appear to correspond to the microscopy details stated in the Methods section.

Upon request, the Authors provided raw data for XRD and FTIR of the catalyst, but the files for XRD and FTIR did not contain metadata that would allow for verification of the veracity of the data. The Authors were also unable to provide a satisfactory response to the concerns raised. Therefore, the Editors no longer have confidence in the presented data.

Ronak Eisavi did not explicitly state whether they agree or disagree with the retraction. Fereshteh Ahmadi did not respond to the Editors’ correspondence about this retraction.

